# Supplementary material for: Effects of a group-based weight management programme on anxiety and depression: A randomised controlled trial (RCT)
Source: PLoS One. 2022 Feb 3;17(2):e0263228. doi: 10.1371/journal.pone.0263228 (PMC8812921; doi:10.1371/journal.pone.0263228)
Supplement: Attachment — Submitted filename: Response to reviewers_final.docx [file pone.0263228.s002.docx]

Dear Professor Ruscica,

Many thanks for allowing us the opportunity to amend our manuscript. Please let the reviewers know that we are very grateful for their time in considering our study and providing valuable comments and suggestions to enhance its readability.

Please find below responses to the comments made by the two reviewers. The changes to the manuscript, highlighted in tracked change function, are uploaded separately as requested, in addition to the manuscript without tracked changes.

*Journal Requirements:*

When submitting your revision, we need you to address these additional requirements.

1. Please ensure that your manuscript meets PLOS ONE's style requirements, including those for file naming. The PLOS ONE style templates can be found at <https://journals.plos.org/plosone/s/file?id=wjVg/PLOSOne_formatting_sample_main_body.pdf> and [https://journals.plos.org/plosone/s/file?id=ba62/PLOSOne_formatting_sample_title_authors_affiliations.pdf](about:blank)

We have followed these guidelines as described.

2. Thank you for stating the following in the Competing Interests section:

‘I have read the journal's policy and the authors of this manuscript have the following competing interests: PA and SAJ are investigators on an investigator-initiated trial funded by Cambridge Weight Plan. PA has done half a day’s consultancy for WW (formerly Weight Watchers). ALA is lead investigator on two publicly funded investigator-initiated trials where the intervention is provided by WW at no cost. None of these activities led to payments to the individuals named.'

Please confirm that this does not alter your adherence to all PLOS ONE policies on sharing data and materials, by including the following statement: "This does not alter our adherence to  PLOS ONE policies on sharing data and materials.” (as detailed online in our guide for authors [http://journals.plos.org/plosone/s/competing-interests](about:blank)).

This has been updated and now reads: 'I have read the journal's policy and the authors of this manuscript have the following competing interests: PA and SAJ are investigators on an investigator-initiated trial funded by Cambridge Weight Plan.PA has presented at two symposia on behalf of the Royal College of General Practitioners that were sponsored by Novo Nordisk. ALA is lead investigator on two publicly funded investigator-initiated trials where the intervention is provided by WW at no cost. None of these activities led to payments to the individuals named. This does not alter our adherence to PLOS One policies on sharing data and materials.’

Funding

The trial was funded by the Medical Research Council National Prevention Research Initiative awarded to AA. Award number MR/J000493/1. The funding partners relevant to this award are (in alphabetical order): Alzheimer’s Research Trust, Alzheimer’s Society, Biotechnology and Biological Sciences Research Council, British Heart Foundation, Cancer Research UK, Chief Scientist Office, Scottish Government Health Directorate, Department of Health, Diabetes 15 UK, Economic and Social Research Council, Health and Social Care Research and Development Division of the Public Health Agency (HSC R&D Division), UK Medical Research Council (MRC), The Stroke Association, Welcome Trust, Welsh Assembly Government, and World Cancer Research Fund.

Weight Watchers donated free behavioural weight management programmes to the NHS to support the trial.

PA, SAJ, and RS are funded by National Institute for Health Research (NIHR) Oxford Biomedical Research Centre and NIHR Oxford and Thames Valley Applied Research Collaboration. LH is funded by an NIHR Academic Clinical Fellowship. There are no specific grant numbers associated with these awards.

If there are restrictions on sharing of data and/or materials, please state these. Please note that we cannot proceed with consideration of your article until this information has been declared.

Please include your updated Competing Interests statement in your cover letter; we will change the online submission form on your behalf.

Please know it is PLOS ONE policy for corresponding authors to declare, on behalf of all authors, all potential competing interests for the purposes of transparency. PLOS defines a competing interest as anything that interferes with, or could reasonably be perceived as interfering with, the full and objective presentation, peer review, editorial decision-making, or publication of research or non-research articles submitted to one of the journals. Competing interests can be financial or non-financial, professional, or personal. Competing interests can arise in relationship to an organization or another person. Please follow this link to our website for more details on competing interests: [http://journals.plos.org/plosone/s/competing-interests](about:blank)

3. We note that you have indicated that data from this study are available upon request. PLOS only allows data to be available upon request if there are legal or ethical restrictions on sharing data publicly. For information on unacceptable data access restrictions, please see <http://journals.plos.org/plosone/s/data-availability#loc-unacceptable-data-access-restrictions>.

In your revised cover letter, please address the following prompts:

a) If there are ethical or legal restrictions on sharing a de-identified data set, please explain them in detail (e.g., data contain potentially identifying or sensitive patient information) and who has imposed them (e.g., an ethics committee). Please also provide contact information for a data access committee, ethics committee, or other institutional body to which data requests may be sent.

b) If there are no restrictions, please upload the minimal anonymized data set necessary to replicate your study findings as either Supporting Information files or to a stable, public repository and provide us with the relevant URLs, DOIs, or accession numbers. Please see <http://www.bmj.com/content/340/bmj.c181.long> for guidelines on how to de-identify and prepare clinical data for publication. For a list of acceptable repositories, please see <http://journals.plos.org/plosone/s/data-availability#loc-recommended-repositories>.

We will update your Data Availability statement on your behalf to reflect the information you provide.

Many thanks for this comment. Our updated data sharing statement reads: ‘Participant consent only allows for data to be used in future research with appropriate ethical approval and data sharing agreements. As such, a managed data sharing process is necessary to ensure that the rights and expectations of participants are protected in line with GDPR. The host institution have an access policy (<https://www.mrc-epid.cam.ac.uk/wp-content/uploads/2019/02/Data-Access-Sharing-Policy-v1-0_FINAL.pdf>). Interested parties can obtain the data for replication or other research purposes by contacting the Principal Investigator Dr Amy Ahern at [ala34@cam.ac.uk](mailto:ala34@cam.ac.uk) or contacting [datasharing@mrc-epid.cam.ac.uk](mailto:datasharing@mrc-epid.cam.ac.uk).’

Reviewers' comments:

Reviewer's Responses to Questions

**Comments to the Author**

1. Is the manuscript technically sound, and do the data support the conclusions?

The manuscript must describe a technically sound piece of scientific research with data that supports the conclusions. Experiments must have been conducted rigorously, with appropriate controls, replication, and sample sizes. The conclusions must be drawn appropriately based on the data presented.

Reviewer #1: Yes

Reviewer #2: Partly

2. Has the statistical analysis been performed appropriately and rigorously?

Reviewer #1: No

Reviewer #2: No

3. Have the authors made all data underlying the findings in their manuscript fully available?

The [PLOS Data policy](http://www.plosone.org/static/policies.action#sharing) requires authors to make all data underlying the findings described in their manuscript fully available without restriction, with rare exception (please refer to the Data Availability Statement in the manuscript PDF file). The data should be provided as part of the manuscript or its supporting information, or deposited to a public repository. For example, in addition to summary statistics, the data points behind means, medians and variance measures should be available. If there are restrictions on publicly sharing data—e.g. participant privacy or use of data from a third party—those must be specified.

Reviewer #1: Yes

Reviewer #2: Yes

4. Is the manuscript presented in an intelligible fashion and written in standard English?

PLOS ONE does not copyedit accepted manuscripts, so the language in submitted articles must be clear, correct, and unambiguous. Any typographical or grammatical errors should be corrected at revision, so please note any specific errors here.

Reviewer #1: Yes

Reviewer #2: Yes

5. Review Comments to the Author

Please use the space provided to explain your answers to the questions above. You may also include additional comments for the author, including concerns about dual publication, research ethics, or publication ethics. (Please upload your review as an attachment if it exceeds 20,000 characters)

Reviewer #1: Thanks for the opportunity to revise this manuscript. The aim of the article was to investigate if a group-based weight management programme worsened anxiety and depression in a group of patients with overweight. The authors concluded that this programme can even ameliorate depressive symptoms in the short-term. These are my main observations about the methods and results of the present research article:

1) Why did the authors select 28 and not 25 as a BMI threshold for overweight?

We did not select 28 as a threshold to define overweight or obesity, but as a threshold where weight loss would benefit health. This is essentially as arbitrary as defining 25 or 30 as a threshold for inclusion, but we thought that 28 included people with excess adiposity without over-including those with a healthy amount of body fat.

2) I am not fully in agreement with some considerations in the introduction. It is well established that overweight and obesity are associated with severity of depressive symptoms (Milaneschi et al., 2019; doi: 10.1038/s41380-018-0017-5). In this sense I wonder if the amelioration of depressive symptoms found in the short term for the group-based weight management programme is not due to a major efficacy in weight loss and in the consequent normalization of underlying biological abnormalities with respect to self-help group (Macchi et al., 2020; doi: 10.1186/s12933-020-01158-. Were there any significant differences in the efficacy on weight loss between groups?

This is an interesting point that we think is worth including. The introduction has been reworded, with this in mind and referencing the papers suggested. This now reads: ‘The effect of obesity on physical health has been well documented [1-5]. People with obesity are at greater risk of poor mental health than those without, but the reasons for this are complicated[6]. Recent evidence suggests that excess weight is associated with severity of depressive symptoms and potential biological mechanisms for this relationship have been explored [7, 8]. Other studies show that intentional weight loss can reduce symptoms of depression, [9, 10] or improve quality of life scores, [11] although this result is inconsistent, with some studies finding no evidence of a relationship between intentional weight loss and health related quality of life scores [12-14]. Further clarification of this relationship is important as there is an outstanding concern that weight loss attempts could worsen mental health [15].’

Yes, there was a difference in the efficacy on weight loss between the groups. This was reported in the main trial (Ahern et al, 2017 <https://doi.org/10.1016/S0140-6736(17)30647-5>), but we agree this result is relevant to this secondary analysis, and so we have included this in the first paragraph of the results section: ‘In the primary study, the mean weight change at 12 months was −3·26 kg (standard error 0·68) in brief intervention, −4·75 kg (0·35) in the 12-week programme, and −6·76 kg (0·42) in the 52-week programme’.

3) The reference (12) is improper as it refers to elderly patients with medical conditions and not affected by overweight.

Many thanks for bringing this to our attention, this reference has been removed and the introduction rewritten as described above.

4) The authors state that they excluded patients with severe mental disorders. However I wonder if some of the included subjects had a psychiatric disorder or were followed up by mental health professionals. Were there patients in treatment with psychotherapy or pharmacotherapy? This is an important confounding factor and it should be at least reported in study limitations if data are not available
Yes, not all participants with a mental health diagnosis were excluded, and some participants had a diagnosed mild or moderate mental health condition with associated raised HADS score. Consequently, a large proportion of people would have been taking medication for mental illness at study start and throughout the study.

Although this presumably would influence scores, it would do so equally in the intervention and control groups. We have now added this to the discussion: ‘Randomisation should distribute covariates equally between groups at baseline, however we cannot exclude post-randomisation bias, such as unequal distribution of pharmacotherapy or psychotherapy between groups after this.’

5) Looking at the results the authors state that mean baseline HADS scores fall in the normal range. However, if you take into account standard deviations, it is likely that some of these patients have clinically significant affective symptoms. Again I wonder if some of these patients received some type of treatment or were followed up by mental health professionals.

Please see our response above. By including people with mental illness, we were able to ensure our results broadly represented the general population of people with overweight and obesity.

6) The authors report that they used mixed regression models for the analysis. Which were the random and fixed effects?

Practice was added as a random effect and all other variables were fixed and we have added that to the description of the analysis.

Reviewer #2: The manuscript entitled ‘Effects of a group-based weight management programme on anxiety and depression: a randomised controlled trial (RCT)’ with the aim to investigate the impact of a group-based weight management programme on symptoms of depression and anxiety compared with self-help in a randomised controlled trial.

This is quite an interesting study, however, the manuscript requires further improvement on the results presentations.

Method
Since the sample size was calculated earlier and published elsewhere it is to be cited.

This has now been updated and is cited.

Completers to be clearly defined.
We agree that this is confusing as currently stated. This has been elaborated on and now reads: ‘We used three models to investigate sensitivity to missing data. As in the primary trial, the main analysis used a mixed model using a missing-at-random (MAR) assumption, and secondary analyses were conducted using multiple imputation and completers only (analysing participants who attended and completed HADS at each specified follow up).’

Results
Table 1, at least 1 decimal point for percentages to be provided.
Agreed, this has now been changed.

Table 2, for CP12 & CP52 (n=1056), what the mean change refers to be clearly denoted. The mean scores at each time period and baseline scores to be provided before deriving the mean change. Likewise with Table 3 prior to adjustment.

We agree this should be changed for table 2. Table 2 has been edited to include mean scores and mean change. This now reads read ‘mean change in HADS from baseline’.

We have given the raw means and change in means in Table 2. However, our analysis focuses on analysing the trial outcome, where it is standard best practice to adjust in the analysis for factors used to stratify the randomisation and, where presenting change scores, adjust for baseline variables. It is not possible to present scores unadjusted for baseline score and it is not desirable to leave out the stratification factors (gender, centre). We trust the referee understands our choice of model selection, which was decided a priori by the design of the trial.

Table 3, technically the p value cannot be zero (to use symbol p <).

Agree, this has now been changed.

Page 15 Paragraph 1 Line 5 for follow-up [0.2 (-0.4, 0.8)]. In Table 3 it was stated [0.2 (-0.3, 0.8)]. Table 3 to be cited in the text.
Thank you for spotting this, we have now corrected it.

Table 3 footnote, for ‘*adjusted for center, gender and baseline depression/anxiety score’ the symbol * to be inserted in the table and a separate adjustment to be denoted separately for each depression and anxiety. Decimal points to be standardized. Word mean to be added to Adjusted difference.

The headings now read ‘Mean adjusted difference’, and the * has been appropriately inserted.

We have ensured all the number of decimal points are consistent. However, we do not believe it would be appropriate to give HADS to 2dp, or p value to 1dp.

Information whether the p value was adjusted to be stated.

There was no adjustment of the p value for multiple testing. This information has been added to the table.

Figure 1 requires revision. Any dropouts to be placed or written outside the box. The time period of assessment to be stated.
Many thanks for this feedback, this has been changed as described.

Figure 2, n to be stated.

We have added ‘Data are mean (standard error) of all measured HADS at each time point (Table 2)’ to direct the reader to the ‘n’ at each timepoint detailed in table 2.

Again, we would like to thank the reviewers for taking time to read our work and provide valuable comments. We hope the above changes are to your satisfaction. Please feel free to contact us should there be a need to clarify any of the comments above.

With many thanks and gratitude.

Dr Laura Heath, Professor Susan Jebb, Professor Richard Stevens, Dr Graham Wheeler, Dr Amy Ahern, Dr Emma Boyland, Professor Jason Halford and Professor Paul Aveyard
