# Supplementary material for: Effects of a group-based weight management programme on anxiety and depression: A randomised controlled trial (RCT)
Source: PLoS One. 2022 Feb 3;17(2):e0263228. doi: 10.1371/journal.pone.0263228 (PMC8812921; doi:10.1371/journal.pone.0263228)
Supplement: Attachment — Submitted filename: Response to Reviewers.docx [file pone.0263228.s003.docx]

*Response to Reviewers*

Many thanks for your further comments about our manuscript. We believe we have fully addressed these points below.

| Please review your reference list to ensure that it is complete and correct. If you have cited papers that have been retracted, please include the rationale for doing so in the manuscript text, or remove these references and replace them with relevant current references. Any changes to the reference list should be mentioned in the rebuttal letter that accompanies your revised manuscript. If you need to cite a retracted article, indicate the article’s retracted status in the References list and also include a citation and full reference for the retraction notice. | Many thanks for highlighting this issue. Reference 16 (Hussain et al, 2020) has been removed from our reference list as this has been retracted. We have replaced this with a current, relevant reference (16):  ‘Gao M, Piernas C, Astbury NM, Hippisley-Cox J, O'Rahilly S, Aveyard P, et al. Associations between body-mass index and COVID-19 severity in 6.9 million people in England: a prospective, community-based, cohort study. Lancet Diabetes Endocrinol. 2021;9(6):350-9. Epub 2021/05/02. doi: 10.1016/S2213-8587(21)00089-9. PubMed PMID: 33932335; PubMed Central PMCID: PMCPMC8081400’  Reference 20 has been updated. This was a preprint reference in the previous submission. This now reads:  ‘Jones RA, Lawlor ER, Birch JM, Patel MI, Werneck AO, Hoare E, et al. The impact of adult behavioural weight management interventions on mental health: A systematic review and meta-analysis. Obes Rev. 2021;22(4):e13150. Epub 2020/10/27. doi: 10.1111/obr.13150. PubMed PMID: 33103340; PubMed Central PMCID: PMCPMC7116866’ |
| --- | --- |
| I agree with the reviewer that high BMI accounted for 4.0 million deaths globally, nearly 40% of which occurred in persons who were not obese. Please address the concern raised. | Please see response below. |
| The authors largely improved their manuscript especially in the part of introduction. On the other hand I am sorry but I still disagree with the authors because a BMI greater than 25 and therefore between 25 and 28 has negative effects on health. This aspect is explicitly reported in available literature (e.g. doi: 10.1056/NEJMoa1614362). This limitation of the study should be clearly mentioned in the text. | Many thanks for your comment. This limitation has now been explored in the discussion and the reference suggested is included in the manuscript.  Discussion, page 12 line 198-201  ‘Another limitation was that only adults with a BMI ≥ 28 kg/m^2^were included. Adults with a BMI between 25-28 kg/m^2^ also have negative health consequences associated with excess weight.(1) Future weight management trials should consider expanding their inclusion criteria to fully incorporate overweight adults.’ |

References

1.	Collaborators GBDO, Afshin A, Forouzanfar MH, Reitsma MB, Sur P, Estep K, et al. Health Effects of Overweight and Obesity in 195 Countries over 25 Years. N Engl J Med. 2017;377(1):13-27.
